# Mitochondrial Transfusion Improves Mitochondrial Function Through Up-regulation of Mitochondrial Complex II Protein Subunit SDHB in the Hippocampus of Aged Mice

**DOI:** 10.1007/s12035-022-02937-w

**Published:** 2022-07-14

**Authors:** A. Adlimoghaddam, T. Benson, B. C. Albensi

**Affiliations:** 1 Division of Neurodegenerative Disorders, St. Boniface Hospital Albrechtsen Research Centre, Winnipeg, MB Canada; 2 Mitrix Bio INC, Pleasanton, CA USA; 3 Department of Pharmacology & Therapeutics, Max Rady College of Medicine, University of Manitoba, Winnipeg, Canada; 4 Department of Pharmaceutical Sciences, College of Pharmacy, Nova Southeastern University, Fort Lauderdale, FL USA

**Keywords:** Mitochondrial dysfunction, Bioenergetics, Complex II, Aging, Age-related disease, Mitochondrial transfusion, Brain, Neuroscience

## Abstract

The mitochondrial theory of aging is characterized by mitochondrial electron transport chain dysfunction. As a hallmark of aging, an increasing number of investigations have attempted to improve mitochondrial function in both aging and age-related disease. Emerging from these attempts, methods involving mitochondrial isolation, transfusion, and transplantation have taken center stage. In particular, mitochondrial transfusion refers to the administration of mitochondria from healthy tissue into the bloodstream or into tissues affected by injury, disease, or aging. In this study, methods of mitochondrial isolation and transfusion were developed and utilized. First, we found a significant decrease (*p* < 0.05) in the expression of mitochondrial complex proteins (I-V) in aged (12 months old) mouse brain tissue (C57BL/6 mice) in comparison to healthy young brain tissue (1 month old). To investigate whether healthy young mitochondria taken from the liver could improve mitochondrial function in older animals, we intravenously injected mitochondria isolated from young C57BL/6 mice into aged mice from the same strain. This study, for the *first time*, demonstrates that mitochondrial transfusion significantly (*p* < 0.05) improves mitochondrial function via the up-regulation of the mitochondrial complex II protein subunit SDHB in the hippocampus of aged mice. This result has identified a role for mitochondrial complex II in the aging process. Therefore, mitochondrial complex II could serve as a putative target for therapeutic interventions against aging. However, more importantly, methods of mitochondrial transfusion should be further tested to treat a variety of human diseases or disorders and to slow down or reverse processes of aging.

## Introduction

Many years ago, it was proposed that mitochondria play a crucial role in aging and age-related diseases. To this end, the targeting of mitochondria has become a critical strategy in several biological processes implicated in aging, such as oxidative stress via reactive oxygen species (ROS) generation, amino acid and lipid metabolism, regulation of apoptosis, and energy metabolism [1–3]. Mitochondria play a key role in energy-demanding brain functions [4]. Oxidative phosphorylation plays an essential role for mitochondria to produce adenosine triphosphate (ATP). A decline in mitochondrial function is associated with a decrease in oxidative phosphorylation capability and an increase in oxidative damage, which leads to aging and age-related diseases [3, 5]. Therefore, there is a strong relationship between energy metabolism and aging and also between energy metabolism and brain disorders.

Several in vitro experiments show that incubation of cells with isolated mitochondria improved total ATP production, respiratory function, and cell proliferation indicating that mitochondrial transfer is beneficial in mitochondria-deficient cells [6–10]. Additionally, several in vivo experiments show that mitochondrial injections protect the heart, liver, and brain tissues from ischemic injury, and also rescue motor dysfunction in Parkinson’s disease and schizophrenia animal models [11–16]. These studies reveal that mitochondrial transplantation can be beneficial in different cell types and disorders [17].

Both the hippocampal and cortical regions of the brain, which are associated with key cognitive functions, are susceptible to aging and thus are susceptible to mitochondrial impairments [18]. Understanding oxidative phosphorylation changes is critical for the designing of novel strategies for therapeutic interventions for aging and even age-related disease. However, our knowledge of how specific mitochondrial mechanisms are affected in aging is currently poorly understood. Moreover, mitochondrial complexes (i.e., I-V) that make up the electron transport chain have been underexplored in a context of aging. Mitochondrial transfusion therapy is a novel strategy that involves the transfer of functional mitochondria into defective brain cells for preventing age-related decline [19, 20]. In this study, we intravenously administrated healthy young mitochondria obtained from the liver of young mice into aged mice to evaluate whether mitochondrial function improves in key aged brain tissues following mitochondrial transfusion. This study provides novel insights for mitochondrial transfusion therapy on aged animals and yields new data for improving hippocampal function via the enhancement of mitochondrial bioenergetics during processes of aging.

## Material and Methods

### Animals

All animal procedures followed guidelines of the University of Manitoba Animal Care Committee, the Canadian Council of Animal Care rules, and the Institutional Animal Care and Use Committee (IACUC) standards. Mice were given ad libitum access to food and water and housed under the standard light*/*dark cycle (12 h light, 12 h dark at room temperature (22 °C)). In this study, we used 1-month-old and 12-month-old male C57BL/6 mice. All C57BL/6 mice were housed and aged in the pathogen-free animal facility at St. Boniface Hospital Research Centre.

### Surgical Procedures

Each mouse was anesthetized by isoflurane inhalation (5% induction, 1.5–5% maintenance). Hair at incision sites was shaved and disinfected with 70% ethanol; eyes were also lubricated. Sterile technique was performed throughout the duration of all procedures according to IACUC requirements. The mouse was placed in dorsal recumbency on a heated table. A small bolster was placed beneath the animal’s head exposing the ventral aspect of the neck. An incision was made from the ramus of one side of the jaw to the tip of the sternum just lateral to the trachea/midline. The mouse was then gently dissected down through the salivary and lymphoid glands, adipose tissue, and fascia to the external jugular vein, which was superficial to most of the neck musculature; also elevated and cleaned the external jugular vein for a distance of 0.5 mm. Then, there was tie off of the distal (cephalic) end of the vein leaving tails 4–5 inches long. Two 1igatures were then placed around the proximal (cardiac) end of the vein. Hemostats were placed on the cephalic suture and one cardiac suture was used to provide gentle counter traction to the vessel. A very small hole was made using vannas scissors. Hemorrhage was controlled with gentle traction on the cephalic ligature ends. The free end of the catheter was inserted into a hole in the vein wall, and was advanced gently to the level of the heart. The cephalic ligature was then tied around the catheter. Furthermore, a small access hole was created on the back of the animal in the mid-scapular region using a hemostat. The vascular access tubing was then led into this pocket and through the hole, allowing the catheter to reach over the neck to the external jugular vein with significant slack to permit free head and neck movement. Next, we attached the button to the tubular end, ensuring a snug fit. The catheter was secured to surrounding tissues using 5–0 absorbable sutures (Oasis Medical, IL), one for the underlying fascia and one to secure the vascular access button to the skin.

### Group Design

The aged mice (12 months old) were randomly divided into three groups. The mice in the first and second groups were injected with the isolated mitochondria (10 mg/kg or 20 mg/kg body weight) via a catheter once every 2 days over the period of 2 weeks (seven total injections). The mice in the third group were injected with the same volume of phosphate-buffered saline-PBS (vehicle). All mice were sacrificed within 24 h after the last day of mitochondrial or PBS transfusion. The mice were assigned (as *n* = 3) in each group for immunoblotting analysis.

### Preparation of Isolated Mitochondria from Mouse Liver

Two C57BL/6 mice of 30 days of age were euthanized by decapitation while under isoflurane anesthetic. Briefly, liver tissues were dissected and washed twice with ice-cold PBS (pH 7.4), and then transferred to a 50-ml falcon tube containing 5 ml ice-cold homogenizing buffer (300 mM sucrose, 10 mM K-HEPES, and 1 mM K-EGTA (pH 7.2)). Then, 250 μl of Subtilisin A (96.61 μM) was added to the homogenate, mixed by inversion, and incubated on ice for 10 min. Then, 5 ml of homogenizing buffer was added to the 40 µm filter and the filter was gently washed by pipetting up and down. Next, a pre-wet 40 µm filter was placed onto a 50-ml falcon tube and the homogenate was filtered into a new 50-ml falcon tube on ice. This step was repeated again with 40 µm and 10 µm filters. The filtrate was then transferred to 1.5-ml Eppendorf centrifuge tubes and centrifuged at 9000 × *g* for 10 min at 4 °C. The supernatant was then discarded; the pellet was resuspended in 1 ml of ice-cold PBS. A final purification step consisted of the sequential passage of the resuspended mitochondria through 0.8-μm pore filters using a 3-ml syringe and transferred to a new 1.5-ml tube.

### Preparation of Isolated Mitochondria from Brain Tissue

Mice were euthanized by decapitation while under isoflurane anesthetic. Brain (hippocampal and cortical) tissues were then removed. Mitochondria derived from transfused and control mice were isolated using the commercial isolation kits (110,170, Abcam, Cambridge, MA, USA) as reported previously [21]. Briefly, hippocampal and cortical tissues were washed twice with 1 × PBS and then homogenized in an isolation buffer. Homogenates were centrifuged at 1000 × *g* for 5 min at 4 °C. Supernatants were centrifuged at 10,000 × *g* for 20 min at 4 °C. Pellets were resuspended with PBS. Furthermore, the protein concentrations of samples were measured utilizing a colorometric DC protein assay kit (Bio-Rad, Hercules, CA, USA) as described previously [22].

### Western Blotting

Samples were added to 4X Laemmli buffer (40% glycerol, 16% SDS, 0.01% bromophenol blue, 20% β-mercaptoethanol, and 0.25 M Tris, pH 6.8) and heated for 8 min at 40 °C. Furthermore, 15 µg of protein from each sample was loaded in each well of a 10% sodium dodecyl sulfate–polyacrylamide (SDS-PAGE) gels (Bio-Rad, Hercules, CA, USA) and at 170 V for 60 min. Following electrophoresis, each was activated using a ChemiDoc imager (Bio-Rad, Hercules, CA, USA). Afterwards, proteins were transferred from gels into the nitrocellulose membranes (Bio-Rad, Hercules, CA, USA) using the Trans-Blot Turbo Transfer System (Bio-Rad, Hercules, CA, USA). Following the transfer, total proteins on the membranes were detected using the ChemiDoc imager. After imaging, the membranes were incubated in TBS-T buffer with 5% bovine serum albumin (BSA) for 1 h at room temperature. All membranes were then incubated overnight on a rocking platform at 4 °C with Total OXPHOS Rodent WB Antibody Cocktail (ab110413, Abcam, Cambridge, MA, USA, 1:1000 dilution). Following primary antibody incubation, membranes were washed with 1X TBS-T buffer (three times; 15 min each) and then incubated with goat anti-mouse IgG (H + L) antibody (Jackson ImmunoResearch Laboratories, West Grove, PA, USA, 1:2000 dilution) prepared in TBS-T buffer with 5% BSA for one and half hours at room temperature. Following secondary antibody incubation, membranes were washed with 1X TBS-T buffer (three times; 15 min each). Then, membranes were treated with enhanced chemiluminescence (ECL) utilizing the Bio-Rad Clarity Weston ECL blotting kit (Bio-Rad, Hercules, CA, USA), and visualized by the ChemiDoc. Relative quantification of OXPHOS protein levels was normalized to total protein as described previously [5, 23].

### Statistical Analysis

The data were analyzed with Student’s *T*-test or with one-way ANOVA, followed by a Tukey post hoc test (GraphPad Prism 6, GraphPad Software) to evaluate the statistical significance of differences between study groups. All values are expressed as the mean ± standard deviation (SD). A difference between or among groups was determined as statistically significant, if *P ≤ 0.05, **P ≤ 0.01, and ***P ≤ 0.001.

## Results

### Effects of Aging on the Expression of Mitochondrial-Associated Proteins in C57BL/6 Brain Tissue

**Fig. 1:**
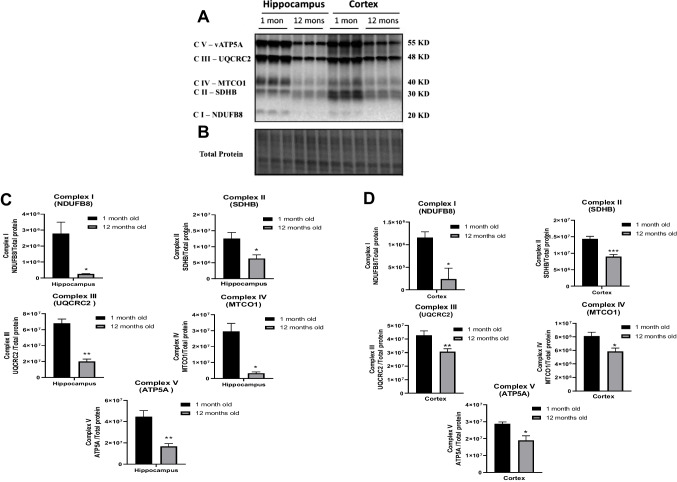
The expression of mitochondrial complex protein subunits declined with age in C57BL/6 brain tissues. Western blot experiments demonstrating relative levels of mitochondrial protein subunits in hippocampal and cortical tissue derived from young (1 month old) and aged (12 months old) C57BL/6 mice. **A**–**B** Representative western blot for NADH dehydrogenase beta subcomplex subunit 8 of complex I (NDUFB8), succinate dehydrogenase subunit B of complex II (SDHB), cytochrome b-c_1_ complex subunit 2 of complex III (UQCRC2), cytochrome *c* oxidase subunit 1 of complex IV (MTCO1), and ATP synthase subunit alpha of complex V (ATP5A). **C** Relative quantification for hippocampal protein levels of complex I-V normalized to total protein. **D** Relative quantification for cortical protein levels of complex I-V normalized to total protein. Results are expressed as mean ± SD of *n* = 3 per group (*P ≤ 0.05, **P ≤ 0.01, ***P ≤ 0.001) analyzed by Student’s *T*-test

In order to evaluate the effect of aging on the mitochondrial respiratory chain, we compared the expression of mitochondrial complex proteins in hippocampal and cortical tissue derived from 1-month and 12-month-old C57BL/6 mice. Interestingly, immunoblotting results showed that the expression levels of NADH dehydrogenase beta subcomplex subunit 8 of complex I (NDUFB8; *p* < 0.05), succinate dehydrogenase subunit B of complex II (SDHB; *p* < 0.05), cytochrome b-c_1_ complex subunit 2 of complex III (UQCRC2; *p* < 0.01), cytochrome *c* oxidase subunit 1 of complex IV (MTCO1; *p* < 0.05), and ATP synthase subunit alpha of complex V (ATP5A; *p* < 0.01) decrease significantly in these brain tissues with age (Fig. 1). Overall, both cortical and hippocampal tissues display significantly higher levels of complex I–V in younger mitochondria compared to the aged ones.

### Effects of Transfused Mitochondria in Mitochondrial-Associated Proteins in C57BL/6 Brain Tissue

**Fig. 2:**
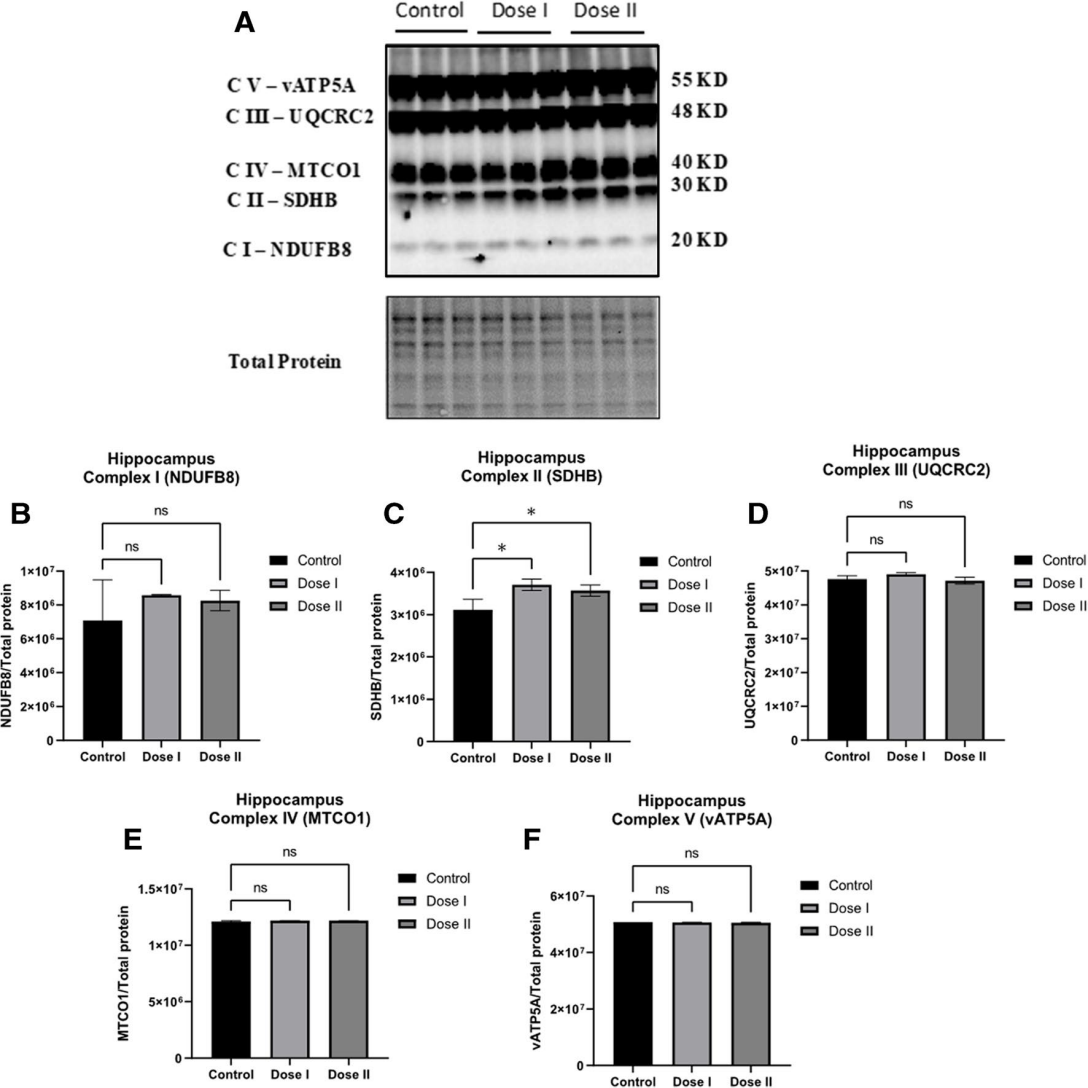
***Transfused mitochondria significantly increased the expression of mitochondrial complex II (SDHB) protein subunit in C57BL/6 hippocampus.*** Western blot experiments demonstrating relative levels of mitochondrial protein subunits in hippocampal tissue of 12 mo old transfused mice (Dose I: 10 mg/kg; Dose II 20 mg/kg). (A) Representative western blot for NADH dehydrogenase beta sub complex subunit 8 of complex I (NDUFB8), succinate dehydrogenase subunit B of complex II (SDHB), cytochrome b-c1 complex subunit 2 of complex III (UQCRC2), cytochrome c oxidase subunit 1 of complex IV (MTCO1), and ATP synthase subunit alpha of complex V (ATP5A). (B-F) Relative quantification for protein levels of complex I-V normalized to total protein. Results are expressed as mean ± SD of n = 3 per group (*P ≤ 0.05) analyzed by one-way ANOVA, followed by Tukey *post-hoc* test

To measure the effect of mitochondrial transfusion on brain mitochondrial function, the expression levels of key mitochondrial subunit proteins were measured in brain tissue utilizing immunoblotting. Mitochondrial transfusion treatment significantly increased (*p* < 0.05) the level of succinate dehydrogenase subunit B of complex II (SDHB) in C57BL/6 hippocampal tissue, indicating that mitochondrial transfusion induces changes in mitochondrial function (Fig. 2). However, levels of NADH dehydrogenase beta subcomplex subunit 8 of complex I (NDUFB8), cytochrome b-c_1_ complex subunit 2 of complex III (UQCRC2), cytochrome *c* oxidase subunit 1 of complex IV (MTCO1), and ATP synthase subunit alpha of complex V (ATP5A) were not significantly different as a function of mitochondrial transfusion treatment in C57BL/6 hippocampus (Fig. 2).

**Fig. 3:**
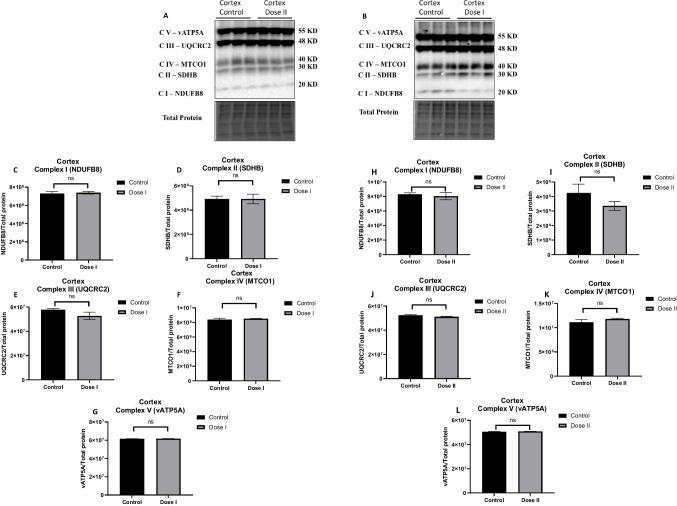
Transfused mitochondria did not alter the expression of mitochondrial complex (I-V) protein subunit in C57BL/6 cortex. Western blot experiments demonstrating relative levels of mitochondrial protein subunits in cortical tissue of 12-month-old transfused mice (dose I: 10 mg/kg; dose II 20 mg/kg). **A**–**B** Representative western blot for NADH dehydrogenase beta subcomplex subunit 8 of complex I (NDUFB8), succinate dehydrogenase subunit B of complex II (SDHB), cytochrome b-c_1_ complex subunit 2 of complex III (UQCRC2), cytochrome *c* oxidase subunit 1 of complex IV (MTCO1), and ATP synthase subunit alpha of complex V (ATP5A). **C**–**J** Relative quantification for protein levels of complex I-V normalized to total protein. Results are expressed as mean ± SD of *n* = 3 per group (*P ≤ 0.05) analyzed by Student’s *T*-test

Immunoblotting results revealed that the expression level of key mitochondrial subunit protein (complex I-V) was not significantly different as a function of mitochondrial transfusion treatment in C57BL/6 cortical tissue (Fig. 3), indicating that transfused mitochondria did not induce changes in mitochondrial function by altering the expression of mitochondrial proteins in C57BL/6 cortex.

## Discussion

Brain adenosine triphosphate (ATP) is generated through oxidative phosphorylation in the mitochondria [24]. Mitochondria derived from aged animals, unlike young animals, cannot produce sufficient supplies of energy as a result of reduced oxidative phosphorylation [25]. Disruptions to mitochondrial oxidative phosphorylation have also been attributed to aging and neurodegeneration [26, 27]. There is still a lack of understanding of how these disruptions may be therapeutically targeted to slow aging, and also to prevent or stop disease progression and improve memory functions. In this study, however, we tested mitochondrial transfusion therapy as a strategy to target biological processes that change during aging. To this end, we looked at mitochondria from both hippocampal and cortical tissue to investigate the effect of mitochondrial transfusion (obtained from the liver).

The methods we utilized in this study for mitochondrial transfusion are straightforward and less expensive. Also, in order to prevent immune responses due to the delivery of exogenous mitochondria to aged mice, we purified mitochondria with several filtration steps. In addition, we monitored the health and safety of the mice during the transfusion procedure, in order to ensure that the method was appropriate and the transfusion of mitochondria did not cause inflammation or death. Likewise, the protocol was optimized to include additional filtration steps to minimize the chance of plugging the catheter with debris, and thus reducing the probability of septic infection. Lastly, the use of filtration eliminates time-consuming centrifugation steps, which offers a quick isolation of purified mitochondria. The key consideration for the efficacy of mitochondrial transfusion is preparing and delivering mitochondria in a timely manner. In this study, the isolation and preparation and measurement of the concentration of mitochondria are timely (within 40 min), which makes the mitochondrial transfusion therapy feasible for clinical practice. The current study does in fact reveal the feasibility of utilizing mitochondrial transfusion as a valued treatment for disorders associated with mitochondrial dysfunction.

The current study reports that mitochondrial transfusion from young mice to aged mice improves the expression of a key mitochondrial protein involved in oxidative phosphorylation. It is suggested that facilitating mitochondrial transfusion and exploring putative mechanisms (e.g., OXPHOS) involved in aging should hasten the application of mitochondria for the treatment of age-related diseases caused by mitochondrial dysfunction.

Previous reports suggest that exogenous mitochondria, following intravenous administration, can cross the blood–brain barrier (BBB) and improve mitochondrial function potentially through the up-regulation of mitochondrial complex II protein subunits in the hippocampal tissue of aged mice. However, the mechanism(s) for penetrating the BBB remains poorly characterized. Our experiments for the *first time* show that young mitochondria may reverse mitochondrial dysfunction potentially by the up-regulation of specific mitochondrial complex protein subunit in aged hippocampal tissues. One recent study by Nitzan et al. [28] in an Alzheimer’s disease (AD) mouse model has also shown that amelioration of mitochondrial dysfunction in the brain was achieved with mitochondrial transfusion. Here they found mitochondrial transfusion increased citrate-synthase and cytochrome *c* oxidase activities relative to untreated AD mice [28]. Several studies reported that the activity of complex II decreases with age in rat heart, lung, kidney, muscle, liver, and brain [29–32]. In a study [33] involving APP/PS1 AD mice, the investigators showed age-dependent decreases in mitochondrial complex II activity starting at 9 months. Recently, our laboratory found that the expression of a complex II subunit significantly decreases in the cortical tissue of 3xTg-AD mice [3]. Other studies indicated that mutations in the subunits of complex II hasten aging and decrease lifespan in *Caenorhabditis elegans* [34] and *Drosophila melanogaster* [35]. Other studies showed age-dependent decreases in mitochondrial complex II activity in human skin fibroblasts and human skeletal muscles [36, 37]. Moreover, in a model for older and younger human skin cells (in terms of telomerase activity), complex II activity was found to be much higher in the younger cells than in the older cells [38]. In addition, a recent study revealed that complex II activity is lower in the skin of naturally aged mice compared to younger mice [39]. These results support our findings and taken together suggest that mitochondrial transfusion may offer a novel therapeutic approach for both aging and age-related diseases, such as AD [37].

In particular, we found that the expression of mitochondrial complex protein subunits decreases in aged brain tissue as compared to younger tissue, which suggests a potential link between complex II and aging. It could be that age-related decline in complex II activity is related to increases in reactive oxygen species (ROS) leakage, which results in oxidative stress and a decrease in normal brain function.

As a next step. future targets should aim to measure oxygen consumption rates and the activity of terminal mitochondrial respiration (cytochrome *c* oxidase). Furthermore, to verify that transplanted mitochondria are in fact internalized into brain cells, transmission electron microscopy (TEM) or light microscopy using fluorescent mitochondrial labels could be utilized, as previous studies have demonstrated the usefulness within cardiac cells [15, 40–42]. In order to determine the activity of mitochondria, the mitochondrial membrane potential could be measured following mitochondrial transfusion using a MitoTracker dye as carried out in ischemic injury studies [43].

In conclusion, our data demonstrate that expression of mitochondrial complex protein subunits decreases as a result of aging and that mitochondrial transfusion can improve specific parameters of mitochondrial bioenergetics in models of aging. In particular, the data indicate that mitochondrial transfusion improves the expression of mitochondrial complex II protein subunits in hippocampal tissue derived from aged mice, which may offer a new therapeutic approach for slowing processes of aging and could have implications for age-related diseases.

## Data Availability

The data and the material supporting this paper are available in the article or are available from the corresponding author upon request.
