# Correction: Mitochondrial Transfusion Improves Mitochondrial Function Through Up-regulation of Mitochondrial Complex II Protein Subunit SDHB in the Hippocampus of Aged Mice

**DOI:** 10.1007/s12035-024-04021-x

**Published:** 2024-02-21

**Authors:** A. Adlimoghaddam, T. Benson, B. C. Albensi

**Affiliations:** 1 Division of Neurodegenerative Disorders, St. Boniface Hospital Albrechtsen Research Centre, Winnipeg, MB Canada; 2 Mitrix Bio INC, Pleasanton, CA USA; 3 Department of Pharmacology & Therapeutics, Max Rady College of Medicine, University of Manitoba, Winnipeg, Canada; 4 Department of Pharmaceutical Sciences, College of Pharmacy, Nova Southeastern University, Fort Lauderdale, FL USA

**Correction to: Molecular Neurobiology (2022) 59:6009-6017**

10.1007/s12035-022-02937-w

After publication of this article [1], the authors became aware of problems with the presentation of Figs. 1 and 2.

In Fig. 1B, the wrong Total Protein image was included. The correct version of the Figure is presented below.
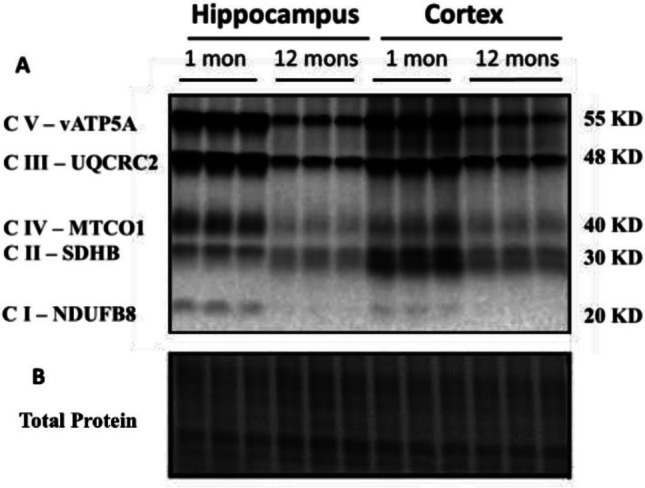


Regarding Fig. 2, authors believe there was a problem with uploading the correct image in the resubmission. Below is correct figure.

**Fig. 2:**
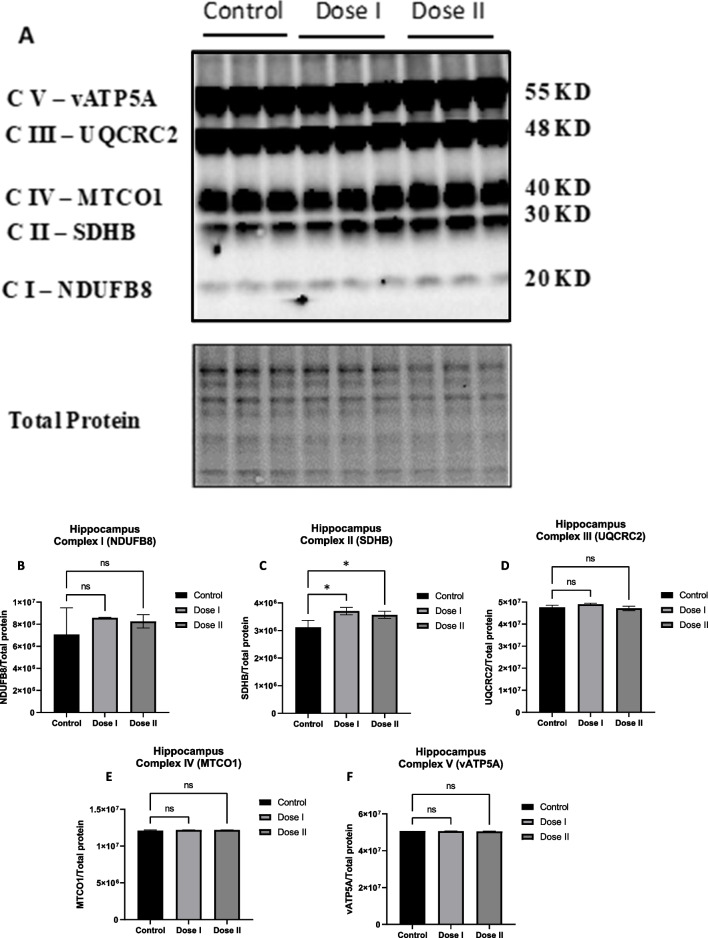
Transfused mitochondria significantly increased the expression of mitochondrial complex II (SDHB) protein subunit in C57BL/6 hippocampus. Western blot experiments demonstrating relative levels of mitochondrial protein subunits in hippocampal tissue of 12 mo old transfused mice (Dose I: 10 mg/kg; Dose II 20 mg/kg). (A) Representative western blot for NADH dehydrogenase beta sub complex subunit 8 of complex I (NDUFB8), succinate dehydrogenase subunit B of complex II (SDHB), cytochrome b-c1 complex subunit 2 of complex III (UQCRC2), cytochrome c oxidase subunit 1 of complex IV (MTCO1), and ATP synthase subunit alpha of complex V (ATP5A). (B-F) Relative quantification for protein levels of complex I-V normalized to total protein. Results are expressed as mean ± SD of n = 3 per group (*P ≤ 0.05) analyzed by one-way ANOVA, followed by Tukey post-hoc test

The original article has been updated to include the correct Figures.

**Competing interests  **Dr. Benedict Albensi is a scientific advisor for Mitrix Bio and the Editor-in-Chief of the Journal of Molecular Neurobiology, but was not involved in handling the manuscript peer review process. The other authors declare that they have no competing interests.

